# Deciphering single- and multi-particle trapping dynamics under femtosecond pulsed excitation with simultaneous spatial and temporal resolution

**DOI:** 10.1038/s41598-022-09251-4

**Published:** 2022-03-30

**Authors:** Anita Devi, Sumit Yadav, Arijit K. De

**Affiliations:** 1 Condensed Phase Dynamics Group, Department of Physical Sciences, Indian Institute of Science Education and Research (IISER) Mohali, Knowledge City, Sector 81, SAS Nagar, Punjab, 140306 India; 2 Condensed Phase Dynamics Group, Department of Chemical Sciences, Indian Institute of Science Education and Research (IISER) Mohali, Knowledge City, Sector 81, SAS Nagar, Punjab, 140306 India; 3 Present Address: Department of Physics, University of Alberta, Edmonton, AB T6G 2R3 Canada

**Keywords:** Nonlinear optics, Optical manipulation and tweezers

## Abstract

Recent theoretical and experimental studies have shed light on how laser trapping dynamics under femtosecond pulsed excitation are fine-tuned by optical and thermal nonlinearities. Here, we present experimental results of trapping of single and multiple polystyrene beads (of 1 μm diameter). We show how integration and synchronization of bright-field video microscopy with confocal detection of backscatter provide both spatial and temporal resolution required to capture intricate details of nonlinear trapping dynamics. Such spatiotemporal detection is promising to have far-reaching applications in exploring controlled laser trapping and manipulations harnessed by optical and thermal nonlinearities.

## Introduction

Optical tweezer is a non-invasive tool that is used for contact-free manipulation of small objects by utilizing the high intensity gradient of a tightly focused laser beam^1^. Over nearly five decades, it revolutionized the exploration of microscopic world with far-reaching applications across disciplines^2^. For trapping of particles with a wide range of sizes, mostly continuous-wave (CW) lasers are routinely used with a few notable exceptions where femtosecond lasers were used^3–6^ which reported that micrometer-sized silica or polystyrene particles show similar (at less power)^3,4^ or less (at high power)^5,6^ trap stiffness compared with CW excitation. In a quest of understanding the fundamental physics underlying these puzzling observations, optical and thermal nonlinearities were shown to modulate the trapping force under femtosecond pulsed excitation both theoretically^7–10^ as well as experimentally^10-12^. As delineated in these works (which focused on optical trapping dynamics under pulsed excitation), at low average power, nonlinear effects are insignificant, hence, trap stiffness is equivalent to CW excitation; however, at high average power, nonlinear optical effects are significant which results in more asymmetry in the axial trapping potential well; while the well becomes steeper before the geometric trap center, it becomes shallower on the other side; equivalently saying, the potential barrier to come out of the potential well or the ‘escape potential’ decreases. Consequently, trap stiffness is less than CW excitation. In addition to mapping this escape potential, experiments revealed a quick ‘adjustment’ of the initially dragged particle characterized by a movement in forward direction which is a result of laser-induced thermal effects in the presence of the particle^10^.

One crucial aspect to disentangle the intricate events in these experiments^10,11^ was to integrate various detection methods (for example, two-photon fluorescence and backscatter detection) using a multimodal optical tweezers set-up^12^ since different methods furnish complementary information. However, in addition to integration, synchronization of different detection modalities is often needed for a complete understanding of trapping dynamics which we present here. In this article, extending our preliminary work^13^, we demonstrate how simultaneous detection of bright-field video microscopy provide a complete understanding of optical trapping dynamics of single and multiple particles inside a nonlinear laser trap created by a femtosecond pulse-train.

**Figure 1:**
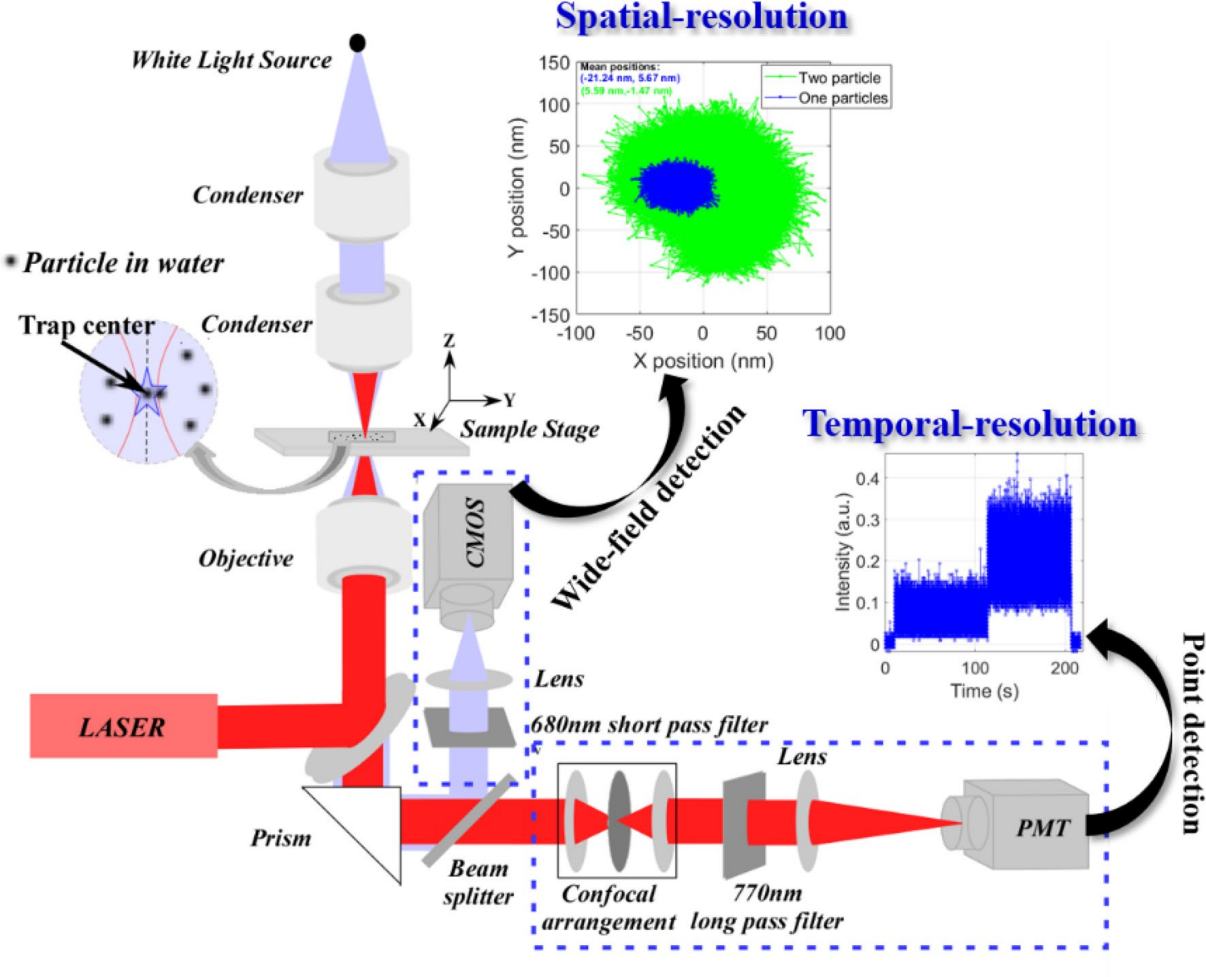
Schematic of the experimental setup.

In Fig. 1, we show the optical layout of the bench-top femtosecond optical tweezer. A detailed discussion of construction and calibration of this apparatus may be found elsewhere^12^. Briefly, following spatial mode clean-up, the expanded laser beam from a Ti–Sapphire oscillator, producing pulses spectrally centered on ~ 800 nm at a repetition-rate of 80 MHz, is used to fill the back aperture of a microscope objective which is used to trap the particles as well as to collect the back-scatter as well as the transmitted white light. The pulse width measured at sample position is ~ 460 fs and the calculated focus spot size (${\omega }_{0}=\frac{0.61\times \lambda }{NA}$) is ~ 500 nm for 800 nm wavelength and 1.3 NA. Diluted and sonicated suspension of 1 µm diameter polystyrene beads in deionized water is put onto a glass coverslip (of 100 µm thickness) and the position of the focal plane from the coverslip surface is kept at ~ 50 μm to avoid any interaction between the particles and glass surface. The transmitted white light and a small fraction (< 5%) of the backscatter pass through a dichroic beamsplitter; further using a 10:90 (R:T) beam-splitter, the scatter and the white light are detected separately and simultaneously. The transmitted white light was imaged onto the CMOS sensor of a camera for video microscopy at a rate of 150 frames-per-second (in ‘cropped’ mode). The reflected scatter was routed to a confocal aperture (using a 200 µm pinhole and an IR filter) for point detection of back-scatter signal using a PMT, recorded by an automated oscilloscope at 400 µs time interval and the data is plotted after 10-point moving averaging of raw data; however, data analysis is done using the raw data (to exclude artifacts associated with averaging^11^) and trap stiffness values are calculated by averaging over more than 10 sets of data.

Although, the sensitivity of bright-field video microscopy on lateral position and that of confocal detection of backscatter on axial position may not seem to complement each other; however, it should be brought in mind that any movement away from the trap center along axial (z) direction renders more room to the particle(s) along transverse (x/y) direction as well because of the converging/diverging trapping beam across the geometric focus. Thus the fluctuations in motions along x-/y- and z-directions are coupled and provide complementary spatial and temporal resolution, respectively, of the same observables.

A comparison of the trapping dynamics under CW excitation and femtosecond pulsed excitation may be found elsewhere^10^. Among the five sequential steps of trapping dynamics (drag, adjustment, equilibration, fluctuation, and ejection), drag and ejection of the particle along axial direction are influenced owing to increased steepness of the axial trapping potential well on one side of the focus (resulting in more dragging force) and increased asymmetry on the other side (resulting in faster ejection) which are due to significant contributions from optical as well as thermal nonlinearities under pulsed excitation compared with CW excitation. Also, the flattening of the well reduces the escape potential, resulting in less confinement time under pulsed excitation.

In Section S1 in the Supplementary Information (SI), we provide a detailed discussion on single-particle dynamics. Earlier, the single-particle dynamics of fluorescent dye coated particle was shown to proceed through sequential steps inside a nonlinear laser trap^10^. These steps are further confirmed here for the uncoated particles. The time constants are found to be similar for both types of particles as both particles are the same in size and were trapped under similar experimental conditions. Interestingly, the confinement time (i.e. the total time over which the particle stays inside the trap, from drag to ejection) for a coated particle is less as compared to an uncoated particle in similar conditions. This is most likely due to destabilization of the trap following absorption-induced heating (i.e. downconversion of photons absorbed by the fluorescent dye molecules, via two-photon absorption, to heat); a detailed discussion and a comparison is shown in Table S1 in SI.

**Figure 2:**
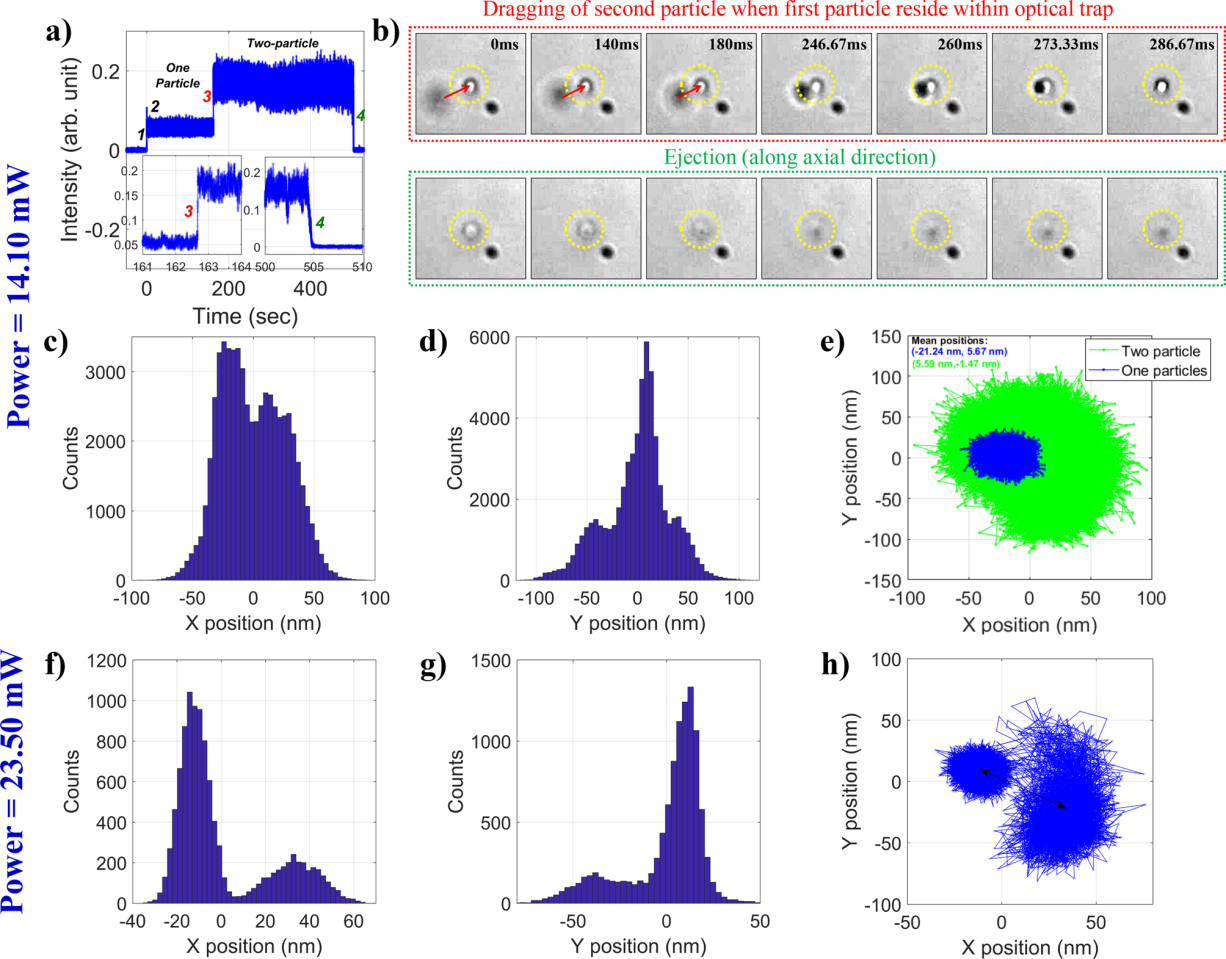
(**a**) Back-scatter signal for two particles, (**b**) transmitted wide-field microscopy images of the particles to map dragging of latter particle when first particle is residing inside the nonlinear optical trap and ejection of both the particles; position distribution along (**c**) x-axis, (**d**) y-axis, (**e**) x–y trajectory; and (**f**) x-axis, (**g**) y-axis, (**h**) x–y trajectory. The data are for confinement of two particles within the nonlinear optical trap at 14.10 mW (first and second row), and 23.50 mW (third row) average power under pulsed excitation.

Figure 2a shows the back-scatter signal for two particles confined in the optical trap. The sudden rise in the back-scatter signal (step no. 3) indicates the dragging of second particle. Noticeably, unlike the single-particle case, this rise is not followed by any drop in the signal that due to adjustment dynamics^10^ (see Section S1 in SI) indicates that the shift in the equilibrium position (due to thermal nonlinearity) is not significant when second particle is dragged. This is because the potential well is already modulated by the presence of first particle. So, further modulation due to dragging of second particle is not significant as it resides away from the trap center. In other words, due to reduced accessible volume, the second particle do not exhibit any adjustment dynamics after initial drag. The integrated signal from both the particles shows fluctuations which corresponds to the lateral shift of the second particle w.r.t the first particle. The confinement time of one particle in absence/presence of the second particle is quite different. Eventually, the ejection dynamics for two particles, can have two possibilities: either both the particles leave the trap (marked as step no. 4 in Fig. 2a) or one particle leaves the trap earlier while another particle stays back. Figure 2b shows the corresponding transmitted wide-field microscopic images of dragging of second particle (which can be dragged from any direction) and ejection of both the particles (which is always along axial direction). The transmitted images give the spatial resolution and by tracking the position of particle(s) in the trap, we get the position distribution and the trajectory of particle. Figure 2c and d show the position distribution of particle(s) along x- and y-axis and Fig. 2e shows the trajectory of particles inside the nonlinear optical trap. The trajectory of single particle shows a clear shift in the mean position on the drag of second particle. A shift in the mean position of the particle’s trajectories is observed (from x = − 21.24 nm, y = 5.67 nm to x = 5.59 nm, y = − 1.47 nm) when the second particle is trapped. However, when two particles are present within the optical trap, fluctuations in the position increase rapidly due to the collision between the particles. Since the particles adjust themselves along axial direction in addition to lateral direction within the trap and stays longer time inside the trap at low average power (14.10 mW), deconvoluting their mean position from video micrographs (which is a collection of –xy projections on CMOS plane) is quite challenging. Also, the position distribution along x- and y-axis for the two particles are also merged in such a way that it becomes difficult to disentangle individual particle’s position distribution; this can be seen from the Fig. 2c–e. Hence, a clear position distribution was not obtained along both x- and y-axis. However, at high average power (23.50 mW), a distinct shift in the particles’ position distribution and localization of individual particles are observed which can be seen from Fig. 2f–h. At high average power, escape potential decreases; consequently, the trap is more asymmetrical in nature for which particles’ confinement time drops from 505 s down to 84 s. Besides, at high average power, potential well broadens, allowing particles to fluctuate more, resulting in a substantial shift in the mean position of the particle’s trajectories. Therefore, the two particles explore different regions (in configuration/real space) but for a short time. Thus, we see two distinct spatial distribution in the trajectory of the particles as shown by a double-sided black arrow in Fig. 2h.

**Figure 3:**
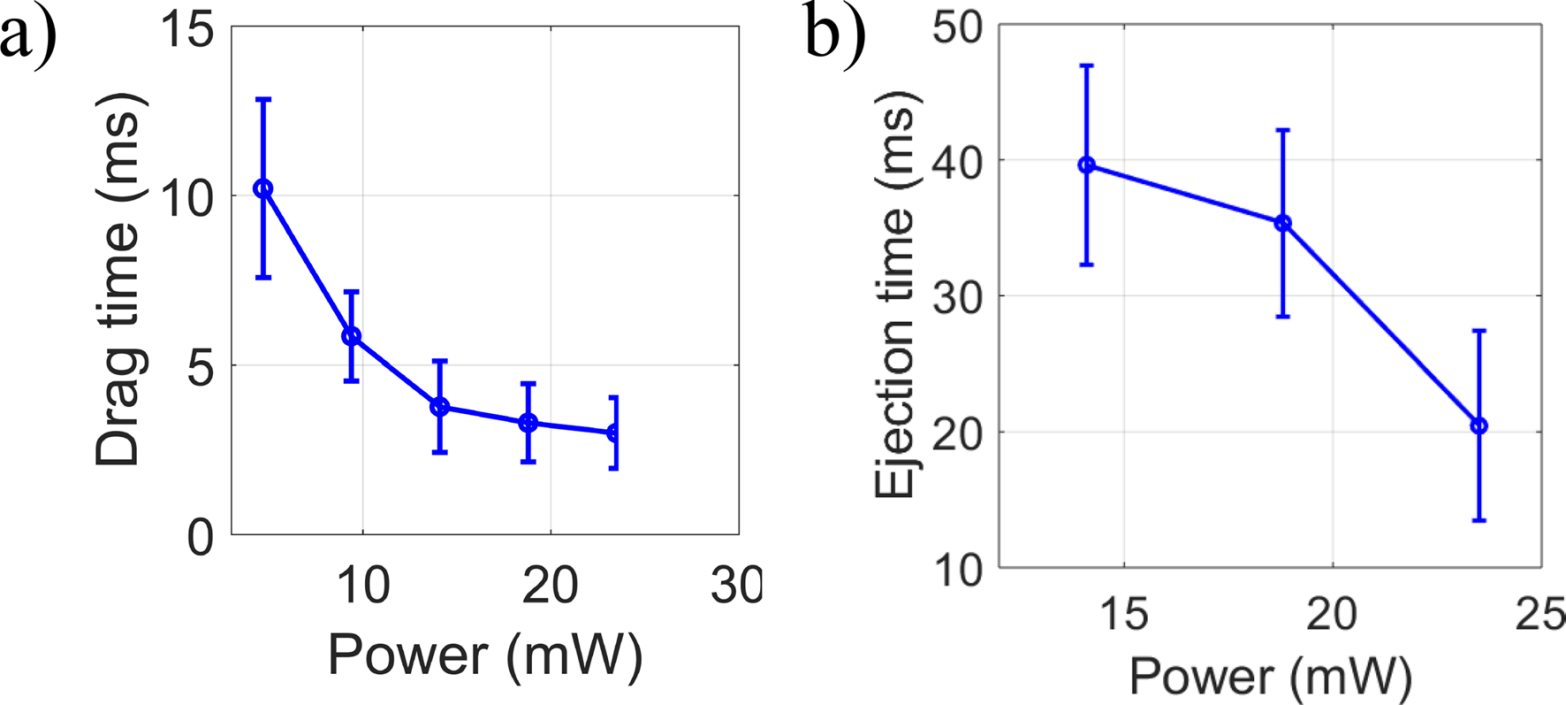
(**a**) Drag, and (**b**) ejection times for the second particle against average power under pulsed excitation.

Figure 3 shows that the time scale of dragging and ejection is in the range of milliseconds and these timescales are further reduced with an increase in power. The drag and the ejection times (as shown in Fig. 2a marked by 3) and the fall (as shown in Fig. 2a marked by 4) of the back-scatter signal, corresponding to the dragging of second particle and axial ejection of both the particles, respectively, are obtained from Sigmoidal fits to the raw data. According to the Nyquist–Shannon sampling theorem, we need resolution in the hundreds of microseconds to identify this scale. Without a high temporal resolution, establishing the drag and ejection time for the second particle is quite difficult; Capturing images at a high frame rate with a standard CMOS camera and interpreting the data is quite challenging. Therefore, we employ a simultaneous point detection approach with a high temporal resolution to capture such fast dynamics^11^.

In Section S2 in the SI, we provide a detailed discussion on dependence of drag and ejection times on laser power. The drag times are different for both the first and second particle as the potential well is different for both the particles. The drag for second particle depends on the modulated potential created by the first particle and the drag time for second particle is more than the first particle at low average power (~ 4.7 mW), which changes with power. This might be due to a significant nonlinear effect at a high power level. The ejection of both the particles is fast as compared to single-particle ejection as two particles possess high collisional energy.

**Figure 4:**
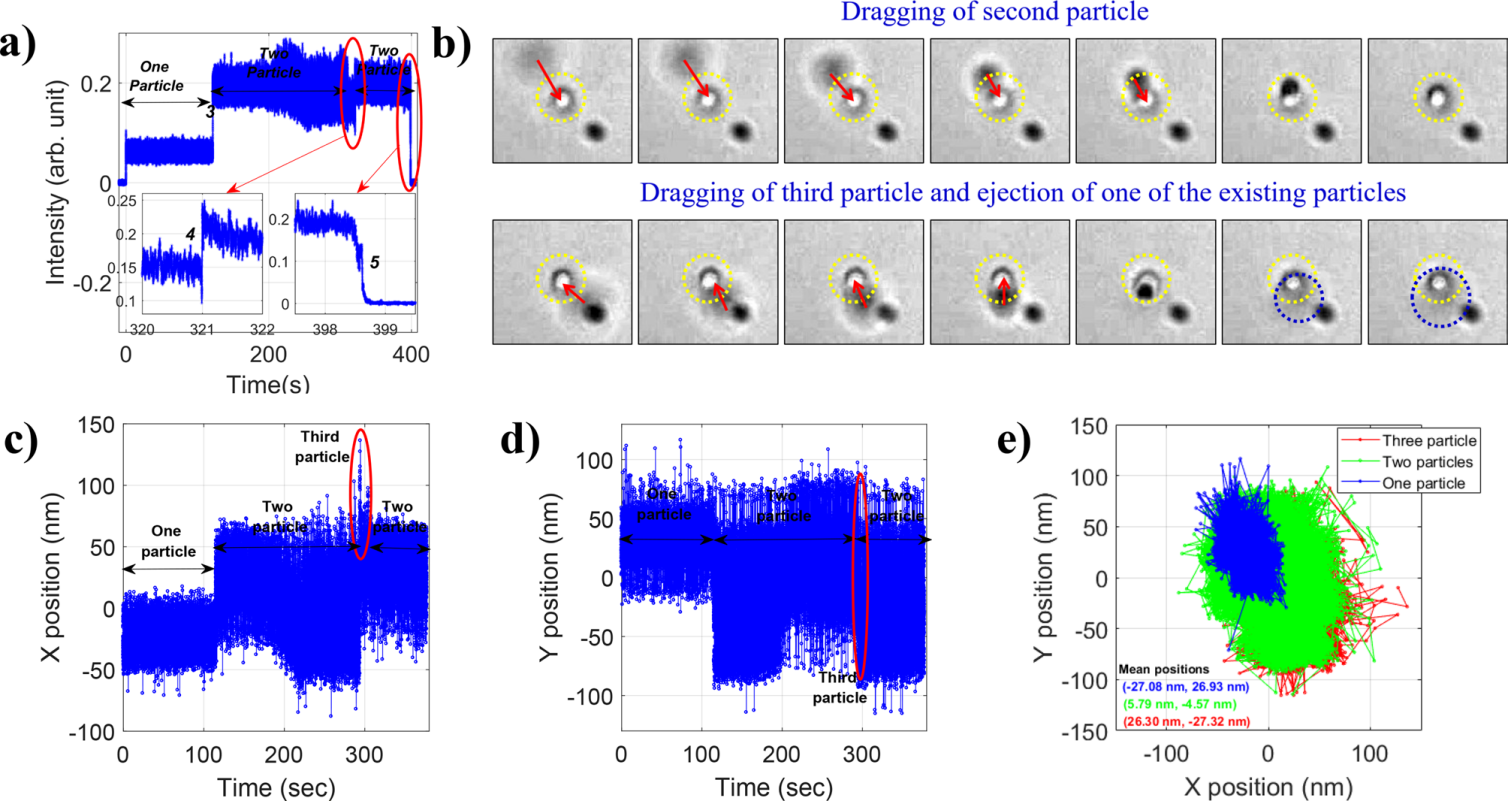
(**a**) Back-scatter signal for multiple (more than two) particles, (**b**) transmitted wide-field microscopy images of the particles to map dragging of second and third particle when first and among one of two particles is residing inside the nonlinear optical trap respectively. Position along (**c**) x-axis, (**d**) y-axis against time, and (**e**) x–y trajectory. The data are for confinement of multiple particle within the nonlinear optical trap at 14.10 mW average power under pulsed excitation.

Figure 4a shows the back-scatter signal (from point detection) from more than two particles being trapped inside the nonlinear optical trap. A sudden rise of the signal indicates the drag of third particle (marked by no. 4) as shown in the zoomed-in trace (the first red circle) in Fig. 4a. The decay in the signal followed by sudden rise indicates the ejection of one of the residing particles. For the particle size and conditions (wavelength of trapping beam, NA of objective, etc.) used in our experiment, no more than two particles can reside in the trap. The zoomed-in trace (the second red circle) in Fig. 4a shows the ejection of both the particles (marked by no. 5). The corresponding transmitted wide-field microscopy images are shown in Fig. 4b. Here, yellow dotted circle represents the trap center, the red arrow shows the dragging direction of second and third particles. From these images, it is very clear that when third particle is dragged, one of the existing particles has to leave the trap which is marked by a blue circle. The ejecting particle is not visualised clearly because ejection is along the axial direction. Figure 4c and d show the plots of x- and y-position of the particles against time, and a shift in the mean position of the particles is observed when second or third particles are dragged within the focal volume. The dragging of third and ejection of one of the existing particles are marked by a red circle. Figure 4e shows the trajectories of the particles here, blue represents the trajectory of single particle, green represents the trajectory after the drag of second particle, and red represents the trajectory after the drag of third particle and ejection of the existing one. The shift in mean positions of the particle trajectories are also mentioned. Similar, behavior is observed at different average powers as well.

To conclude, we have shown how synchronized spatiotemporal detection provides a vivid picture of nonlinear laser trapping dynamics of single and multiple particles under femtosecond pulsed excitation.

## Supplementary Information

Supplementary Information.

## Data Availability

The data that support the findings of this study are available within the article and its Supplementary Material.
