# Supplementary material for: Deciphering single- and multi-particle trapping dynamics under femtosecond pulsed excitation with simultaneous spatial and temporal resolution
Source: Sci Rep. 2022 Mar 30;12:5373. doi: 10.1038/s41598-022-09251-4 (PMC8967920; doi:10.1038/s41598-022-09251-4)
Supplement: Supplementary file 1 — Supplementary Information. [file 41598_2022_9251_MOESM1_ESM.pdf]

Deciphering single- and multi-particle optical trapping dynamics under femtosecond pulsed excitation with simultaneous spatial and temporal resolution

Anita Devi[a, §], Sumit Yadav[a], and Arijit K. De[b]*

Condensed Phased Dynamics Group, [a]Department of Physical Sciences and [b]Department of Chemical Sciences, Indian Institute of Science Education and Research (IISER) Mohali, Knowledge City, Sector 81, SAS Nagar, Punjab 140306, India.

[§]Present affiliation: Department of Physics, University of Alberta, Edmonton, AB T6G 2R3, Canada.

*akde@iisermohali.ac.in

S1. Single-particle dynamics:

The single-particle dynamics is discussed in reference 1 for fluorescent coated particles using the point detection method. Here, we correlated point detection data with wide-field image data for clear visualization of dynamics of an uncoated polystyrene beads. Figure S1a shows that when the particle is confined within the nonlinear optical trap, it follows five sequential steps: drag (marked by 1), adjustment (marked by 2), equilibration (marked by 3), fluctuation (marked by 4), and ejection (marked by 5). The adjustment dynamics might be fast enough that it is difficult to observe in wide-field mode at low frame rate. Figure S1b shows here that at a high frame rate (150 fps), adjustment can be captured in bright field image. The adjustment time is expected to decrease with decreasing particle size and difficult to observe for a few hundreds of nanometer particle size [2] and it depends on the viscosity of the medium (a more viscous medium can slow down the particle [1]). Also, the position of global minima along the axial direction is not fixed for similar excitation condition because it depends on many factors including particle size, the wavelength of trapping beam, refractive index (RI) of the surrounding medium, linear and nonlinear RIs of the particle, numerical aperture (NA) of focusing objective, etc. Figure S1b shows the corresponding transmitted wide-field microscopic images of drag, adjustment, and ejection dynamics. To calculate the trap stiffness of the trapped particle, we calculat the position distribution of particles along x- and y-axis which is fitted with a normal distribution (Figures

S1c-d). Figure S1e shows a single particle's trajectory when it is confined within a nonlinear optical trap corresponding to the back-scatter signal shown in figure S1a. Figures S1f-g show the position of the particle along x- and y-direction against time that gives the information of lateral shift (along a single direction) of particle's position. The trap stiffness k_x, k_y and k_r for the confined particle are 40 pN/μm, 29 pN/μm, and 24 pN/μm, respectively at 14.10 mW average power. The difference in trap stiffness along x- and y-axis is most likely due to an asymmetry in the beam profile or slight slope/tilt in the sample stage [3]. Figure S1h shows the trap stiffness over time, the trap stiffness measured over 20 sec time window to estimate the particle dynamics when the particle leaves the trap. This indicates the equilibration and fluctuation dynamics of the trapped particles. During the equilibration dynamics, for initial time windows, the trap stiffness is approximately constant and then gradually start decreasing when particle start leaving the trap (fluctuation dynamics). Eventually, when particle leaves the trap, fluctuation is more along x- and y- direction which results in decreasing the trap stiffness.

Under pulsed excitation, the nonlinear trapping potential and particle dynamics strongly depend upon the average power [1], pulse width, pulse repetition rate and wavelength of the trapping beam. Thus, to generalize the particle dynamics under pulsed excitation, we have studied how confinement, drag, adjustment, ejection, and trap stiffness changes at different average power. Figure S2 shows the time constants corresponding to the particle dynamics, maxima of back-scatter signal (immediately after the particle is dragged) and the trap stiffness against average power. It can be seen that all the five sequential dynamics of coated and uncoated particles are same and the time constant against average power follows a similar trend (comparing with figure S2a-e and figure 3k-o in reference 1). A comparison of time constants of both types of particles (dye-coated as well as un-coated) shown in table S1. The confinement time for coated particle is less as compare to uncoated particle due to the thermal dissipation of the absorbed energy absorbed photons (by the dye molecus coated on the particle) while time constants for other dynamics do not show any significant difference. Due to this, coated particles get more continuous thermal kicks from the environment, which increases the particles' fluctuations along radial and axial directions inside the optical trap as compared to uncoated particles (figure S2a), which can be seen by comparing figure 3n of reference 1 and figure S2a or in table S1. Figure S2f shows the trap stiffness against average power, and it can be evident that as power increases, the lateral trap stiffness increase monotonically. At low average power, the

trap stiffness along the x-axis and y-axis is approximately equal. However, it deviates as average power increases due to the significant contribution of nonlinear effects. That could be one of the reasons that we observe a significant difference in trap stiffness along x- and y- axis for figure S1h.

S2. Two-particle dynamics:

The drag time for the second particle decreases with increasing average power because the steepness of the potential increases significantly. Similarly, ejection time also decreases with increasing average power because the asymmetry of the trap increases with average power, so at high average power, the curvature of the potential well flattens along the direction of ejection. Noticeably, at low average power particle confinement time is so long that detecting dynamics for second particle is bit difficult because the beads tend to aggregate over time. At very high average power, the escape potential is not enough to confine two particles. Consequently, as soon second particle is dragged, both particles leave the trap together.

S3. References:

1. A. Devi, S. Yadav, and A. K. De, "Dynamics of a dielectric microsphere inside a nonlinear laser trap," Appl. Phys. Lett. **117** (16), 161102 (1-6) (2020).

2. A. Devi, and A. K. De, "Simultaneous detection of two-photon fluorescence and back-scattered signal of optical trapping of dielectric nanoparticles under femtosecond pulsed excitation," J. Nanophotonics **13(2)**, 020501(1-8) (2019).

3. A. Devi, and A. K. De, "A table-top compact multimodal nonlinear laser tweezer," Opt. Commun. **482,** 126440 (2020).

Figures:

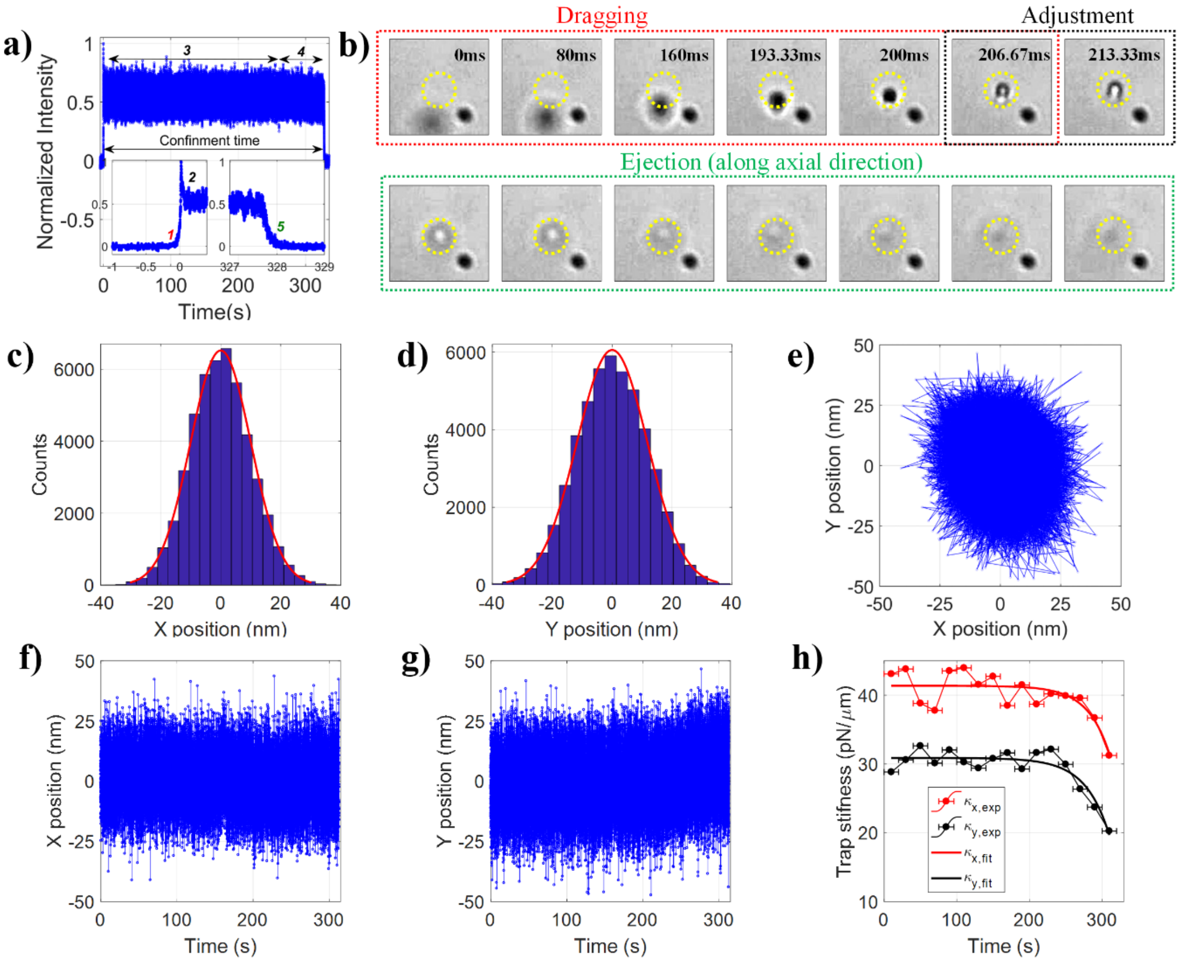

Fig. S1. a) Back-scatter signal, and b) transmitted wide-field microscopy images of the particle to map dragging, adjustment and ejection; position distribution along c) x-axis, d) y-axis, e) x-y trajectory; variation of f) x-position against time, g) y-position against time, and h) evolution of trap stiffness evaluated at every 20 sec time interval. All data are for a single confined particle within nonlinear optical trap at 14.10 mW average power under pulsed excitation.

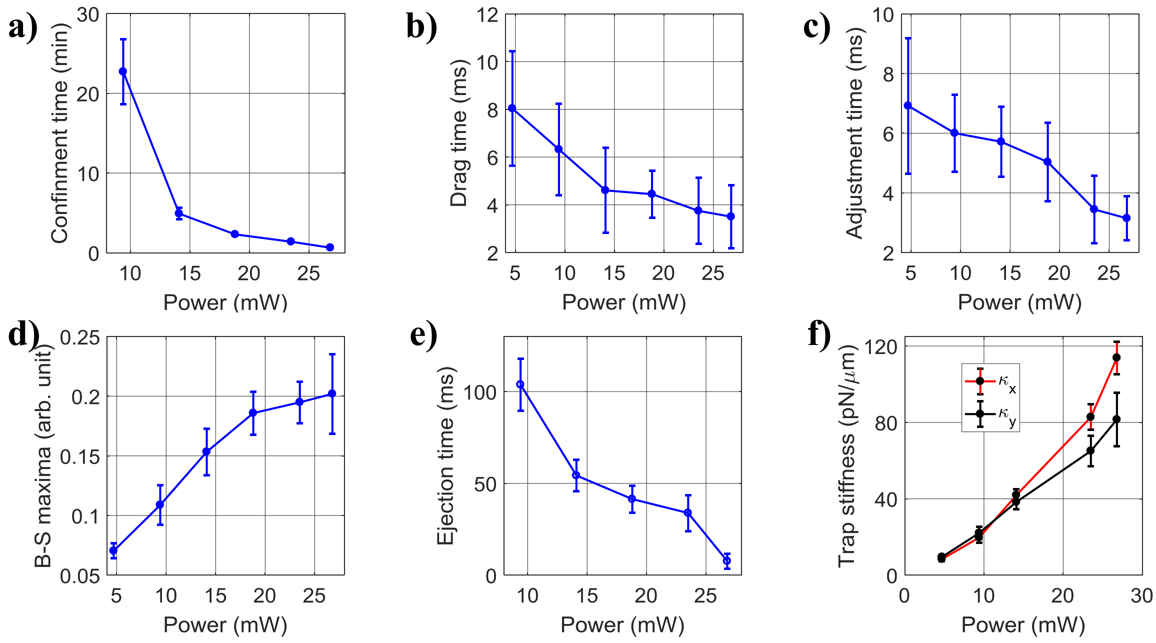

Fig. S2. a) Confinement time, b) drag time, c) adjustment time, d) peak intensity, e) ejection time, and f) trap stiffness against average power for a single confined particle within nonlinear optical trap under pulsed excitation.

Table:

Table S1. Time constants corresponding to drag, adjustment, and ejection dynamics and confinement time for coated and uncoated particle.

Un-coated particle					Coated particle [1]			
Power (mW)	Confinement time (s)	Drag time (ms)	Adjustment time (ms)	Ejection time (ms)	Confinement time (s)	Drag time (ms)	Adjustment time (ms)	Ejection time (ms)
4.70	---	5±2.3	6.9±2.2	---	---	---	---	---
9.40	1362.5±244.5	6.3±1.9	6±1.2	103.7±14.1	558.1±28.9	---	---	164.7±22.7
14.10	294.5±43.8	4.6±1.7	5.7±1.1	54.2±8.6	169.1±25.9	5.3±1.9	5.6±1.6	82.9±40.2
18.80	138.4±9.7	4.4±0.9	5±1.3	41.4±7.3	102.4±23.9	3.8±2.4	5.1±1.5	76.8±11.1
23.50	83.2±3	3.7±1.3	3.4±1.1	33.8±9.7	57.9±3.7	2.4±1	4.6±1.3	70.2±24.3
